# Tyrosinase Inhibitors Among Flora of Lubelskie Region—Application of Bio-Chromatographic Approach and Zebrafish Model in Bioactivity Screening of Plant Material

**DOI:** 10.3390/molecules30091979

**Published:** 2025-04-29

**Authors:** Kamila Kusio-Targońska, Nataliia Kosheva, Krzysztof Kamil Wojtanowski, Katarzyna Gaweł-Bęben, Dimitris Beis, Wirginia Kukula-Koch

**Affiliations:** 1 Department of Pharmacognosy with Medicinal Plants Garden, Medical University of Lublin, 20-093 Lublin, Poland; kamila.kusio-targonska@umlub.pl (K.K.-T.); krzysztofkamilw@gmail.com (K.K.W.); 2 Department of Experimental and Clinical Pharmacology, Medical University of Lublin, 20-090 Lublin, Poland; nataliia.kosheva21@gmail.com; 3 Department of Cosmetology, The University of Information Technology and Management in Rzeszow, 35-225 Rzeszow, Poland; kagawel@wsiz.edu.pl; 4 Laboratory of Biological Chemistry, Faculty of Medicine, School of Health Sciences, University of Ioannina, 45110 Ioannina, Greece; dbeis@uoi.gr

**Keywords:** chamomile, cosmetic properties, bioassays, HPLC-MS, plants

## Abstract

The whitening potential of natural products is commonly assessed through spectrophotometric assays that colorimetrically measure the inhibitory effects on tyrosinase, a key enzyme in pigment formation. However, these assays fail to provide evidence about the input of individual components into the total activity of a mixture like plant extracts. This study introduced chromatographic methods to identify active natural products without isolating them from their mixtures. In this study, various plant extracts of differing polarities (EtOH, 50% EtOH, and HOH) from species growing in the Lubelskie region of Poland were evaluated for their ability to inhibit tyrosinase. The most active extract identified through spectrophotometric assays was a 50% EtOH extract from *Matricaria recutita* L. (*Chamomilla recutita* (L.) Rauschert). Subsequent HPLC-MS analysis allowed for the identification of several active compounds from different classes, including organic acids, glycosylated phenolics, and phenolic acids that interacted with the enzyme. The bioactivity of individual components was confirmed through classical spectrophotometric assays, highlighting ferulic acid (IC_50_ = 0.484 µM), quinic acid (IC_50_ = 22.90 µM), and citric acid (IC_50_ = 24.18 µM) as three representatives of different classes of molecules with inhibitory potential. Furthermore, the whitening capacity of the chamomile extract was investigated in a zebrafish model, demonstrating effective pigmentation inhibition in *Danio rerio* larvae and validating the proposed chromatographic approach.

## 1. Introduction

The cosmetic industry has emerged as one of the most dynamically developing sectors in recent years. The European cosmetic market represents a value of EUR 96 billion (with the UE accounting for EUR 81 billion), comprising a total of 8540 small and medium-sized enterprises (SMEs), and employing nearly 3,033,000 individuals, including 30,040 scientists and 740,191 life science students. According to the organization Cosmetics Europe, 72% of European consumers consider cosmetics to be important or very important in their daily lives, with an average usage of seven different cosmetic products each day and thirteen on a weekly basis. These figures are even higher among women and younger demographics. While the primary reasons for using cosmetics include personal hygiene, well-being, and protection of the skin, hair, and nails, it is noteworthy that 70% of European consumers report using cosmetics to enhance their self-confidence [1].

An increasing interest in a healthy lifestyle is evident not only in dietary choices but also in cosmetics and medicine. Consumers are becoming more aware of health impacts, leading to a heightened demand for maintenance products based on natural ingredients. Presently, most consumers prefer natural products over their synthetic counterparts. In recent years, there has been a resurgence of plant-derived ingredients sourced from organic farms in both the medical and cosmetic fields. The term “naturally derived” resonates strongly with consumers and is often perceived as a guarantee of robust sales. This trend has encouraged manufacturers to favor enriched extracts, fractions, or even single purified components over crude extracts, with the aim of enhancing cosmetic efficacy. Resveratrol, caffeine, epigallocatechin gallate, and gallic acid are just a few examples of purified natural substances registered in the cosmetic ingredient database [2].

Currently, ingredients utilized in cosmetic formulations are expected to perform multiple functions providing protection against harmful environmental factors, such as air pollution, ultraviolet radiation, and varying climate conditions (e.g., wind and temperature). Ultraviolet radiation (UVR) is one of the most detrimental elements affecting the skin, as it stimulates the development of various pathological conditions. Excessive exposure to UVR poses significant health risks, including the development of skin cancer, as well as aesthetic issues such as pigmentation disorders [3].

In light of ongoing environmental changes, there is a growing demand for cosmetic ingredients that offer both antioxidant and skin-brightening effects. Skin discoloration has become an increasingly significant aesthetic and health concern, arising from disruptions in melanin synthesis and its uneven distribution in the skin [4].

Hyperpigmentation, commonly referred to as discoloration, manifests in various unesthetic forms, including primary eruptions, most frequently appearing as spots. Melanin, a natural pigment synthesized in melanocytes, protects the skin from the harmful effects of solar radiation by absorbing UVA and UVB rays and neutralizing free radicals, originating from the amino acid tyrosine. Melanin is present in varying degrees in human and animal skin, and it is responsible for the coloration of skin, hair, and eyes. Any abnormalities occurring during melanocytophagocytosis and pigment transport can lead to dyschromia, which is challenging to treat and may result in the formation of persistent pigmentation spots [5].

Over the past few decades, a variety of tyrosinase inhibitors, both natural and synthetic, have been identified. Some of the well-known inhibitors include hydroquinone, azelaic acid, retinoids, arbutin, derivatives of ascorbic acid, kojic acid, polyphenolic compounds, glycolic acid, and resveratrol. However, certain commonly used inhibitors, such as kojic acid, arbutin, hydroquinone, and vitamins C and B3, have been associated with undesirable side effects, including skin irritation, dryness, allergic reactions, but also cytotoxicity, dermatitis, and even tumorigenesis [6]. Therefore, it is imperative to identify and characterize safe and effective pharmacological inhibitors of tyrosinase.

In the search for new ingredients for cosmetic formulations, modern analytical techniques play a pivotal role. Among the employed techniques for assessing whitening properties, spectrophotometric measurement methods make a substantial contribution. In studies on the cosmetic significance of samples, ready-made assay protocols are conducted using 96-well plates, and the tyrosinase-inhibitory potential against fungal, murine, or human tyrosinase is determined for the entire dosed sample. This approach facilitates the comparison of the efficacy of various formulations in inhibiting tyrosinase activity.

In the case of plant extracts that are rich mixtures of organic substances, the percentage of enzyme inhibition obtained from spectrophotometric studies relates to the total activity of the entire sample. The determination does not allow for the observation of the behavior of individual active components, which is of significant importance in the development of new cosmetic formulations. Therefore, recent years have brought the elaboration of new approaches aiming at the integration of chromatographic methods with a bioassay. The approach, in which the reaction mixture is analyzed using a liquid chromatograph coupled with different detectors (e.g., UV detectors or mass spectrometers), may facilitate the identification of individual components or ions that interact with the enzyme without the need for their isolation from the mixture, thereby saving both time and reagents.

So-called on-flow assays represent a comprehensive analytical approach involving analyte separation, purification, identification, and even quantification within a single, continuous, and automated process. Among the tested approaches, two distinct on-flow methodologies applicable for screening assays are distinguished: activity-based methods and affinity-based methods. The former assays offer direct insights into the interactions between potential ligands and the catalytic site of the enzyme when the biological target has a well-defined catalytic function. Conversely, affinity-based assays provide a broader perspective, enabling rapid evaluation of ligand interactions with the entire structure of the biological target, which is the case in this study. This is particularly advantageous when the goal is to identify a ligand from a complex mixture [7]. The simplest analytical configuration for on-flow screening using High-Performance Liquid Chromatography (HPLC) involves direct coupling of the enzyme conjugate to a selective detector, such as a mass spectrometer, facilitating the differentiation of enzymatic products, substrates, or inhibitors without the need for extensive purification steps. Recent studies have demonstrated the successful application of these methodologies, including simultaneous assays involving different enzymes, thereby validating their efficacy in ligand recognition and screening. Notable examples of affinity-based methods include the search for xanthine oxidase inhibitors in *L. macranthoides* and *S. miltiorrhiza* extracts [8,9]; the screening of maltase, invertase, and lipase conducted by Tao et al. [10]; and the investigations into acetylcholinesterase inhibitors [11].

The aim of this work was to draw attention to analytical tools that can sensitively screen plant extracts and other mixtures of organic compounds for their potential cosmetic significance without the need for prior isolation.

To develop the methodology, plant species extracts from the Lubelskie region were used. This particular area, located in the southeast of Poland, is distinguished by exceptional natural environmental conditions and ecological purity. Due to its diverse climatic and soil conditions, it is rich in a variety of medicinal plants, both wild and cultivated by local farmers and gardeners. It is worth noting that the Lubelskie region is a leader in herbal production in Poland. The most popular medicinal plants grown and found in this region are also some of the oldest medicinal herbs used by humankind [12]. Among the representatives of the local flora, this study tends to investigate lesser-explored species for their cosmetic significance, specifically focusing on the search for effective tyrosinase inhibitors.

With the ongoing development of analytical methods, characterized by increasing selectivity and sensitivity, it is essential to emphasize that traditional fractionation techniques, which are often time-consuming and resource-intensive, should be reconsidered.

New methodologies should prioritize initial screening processes that can rapidly identify promising candidates, followed by the isolation and activity assessment of individual molecules only when warranted by the screening results. This shift in approach enhances efficiency and optimizes resource allocation in research and development.

Also, to validate the accuracy of the proposed methodology and the effectiveness of the screening process, the results obtained from the tyrosinase inhibition assays and bio-chromatographic analysis conducted in vitro are compared with findings from an in vivo assay using zebrafish larvae. This comparison aims to demonstrate the physiological effects of the screened compounds within a living organism, thereby providing a comprehensive understanding of their bioactivity and potential for cosmetic applications. By establishing a correlation between in vitro and in vivo results, this study seeks to reinforce the reliability of high-throughput screening techniques in the identification of bioactive compounds and their subsequent application in the cosmetic industry.

## 2. Results

### 2.1. Extraction and Fingerprinting of the Obtained Extracts

The *Chamomilla recutita*, *Calendula officinalis*, *Taraxacum officinale*, *Rosa canina*, *Sambucus nigra*, *Crataegus monogyna*, *Urtica dioica*, or *Equisetum arvense* analyzed in this study formed a collection created from the local cultivators, also in consideration of their diverse compositional portfolio and a wide range of secondary metabolites types identified in the extracts. The performed study led to the creation of a kind of extract library. Three extracts of different polarity were obtained from every selected plant species, chamomile, marigold, dandelion, rosehip, elderberry, hawthorn, nettle, and horsetail, as presented in Table 1. The obtained extracts were assessed for their tyrosinase inhibitory potential.

**Figure 1:**
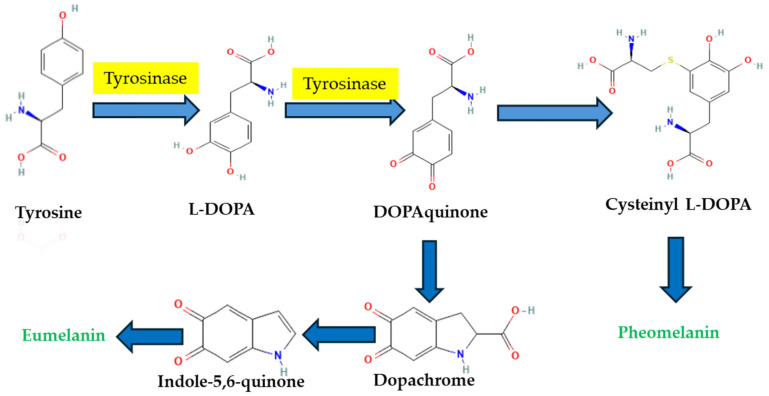
The pathways for melanin formation including the role of tyrosinase.

Tyrosinase is the most important enzyme engaged in the melanin formation—a catalyst of the two initial sequences in melanin biosynthesis (Figure 1). During the process of melanogenesis, the enzyme tyrosinase primarily plays a crucial role by catalyzing the conversion of L-tyrosine to 3,4-dihydroxy-L-phenylalanine (L-DOPA) through hydroxylation, which is followed by the oxidation of L-DOPA to quinone DOPA [5]. Since tyrosinase is the rate-limiting enzyme in melanogenesis, considerable research has focused on the discovery and development of inhibitors that target this enzyme for both therapeutic and cosmetic uses.

As shown in Table 1, the tested samples were characterized by differentiated whitening properties. Among the extracts, the 50% EtOH extract from chamomile showed the highest inhibitory potential against mushroom tyrosinase, whereas water extract from *Taraxacum officinale* was characterized by the weakest activity (75.8% and 27.7%, respectively).

Chamomile has been proven to exhibit whitening properties in previous studies. Research performed by Jae-Bum Jo et al. [13] showed that the tyrosinase inhibition of *M. chamomilla* was dependent on the type of extract tested. The water extract was ineffective, but 60% ethanol extract was shown as a tyrosinase inhibitor with a concentration-dependent action. In another study, 50% EtOH extract was reported as the strongest extract, whereas 96% EtOH did not recover tyrosinase inhibitory constituents [14].

Interestingly, the analysis of the data obtained in this study showed that some of the tested extracts intensified the potential of tyrosinase. Hence, they may exhibit an opposite effect and intensify the pigmentation process. This was the case for *Rosa canina*, *Sambucus nigra*, *Crataegus* *monogyna*, *Urtica dioica*, and *Equisetum arvense* extracts.

Due to the highest tyrosinase inhibitory potential of chamomile, its ethanol/water extract was selected from all tested samples and used for further fingerprinting studies by HPLC-MS methodology and for trials in the development of the bio-chromatographic approach in the search for potential tyrosinase inhibitors.

### 2.2. Qualitative Fingerprinting of Chamomilla recutita Extract by HPLC-ESI-QTOF-MS/MS

**Figure 2:**
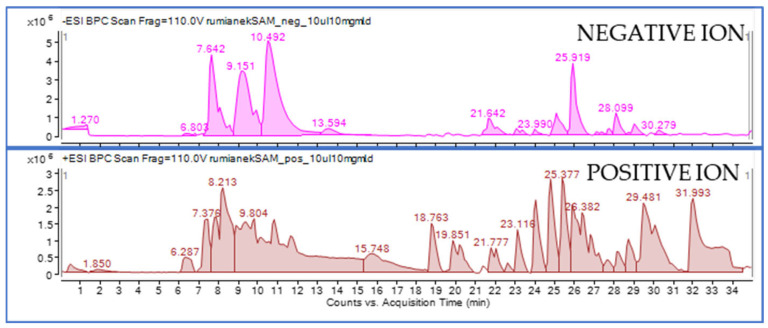
Mass chromatograms recorded in negative and positive ion modes for the total 50% EtOH extract from chamomile.

The qualitative fingerprinting accomplished using an HPLC-ESI-QTOF-MS/MS instrumentation was later performed on the 50% EtOH extract from chamomile as the most active extract, indicated by the spectrophotometric assay. Figure 2 displays the mass chromatograms obtained in both negative and positive ion modes that were recorded for the total 50% EtOH extract of chamomile and shows a multitude of *m*/*z* features distributed in both chromatograms.

The table below (Table 2) contains the tentatively identified components of the chamomile 50% EtOH extract, whereas the spectral data are presented in the Supplementary Materials Table S1 and Figure S1.

The fingerprints of chamomile exhibit assigned signals that have been characterized in the scientific literature [15,16,17]. The results presented in this paper are similar to the previous report by Yassin Ismail et al. in terms of the main classes of identified compounds, with slight differences within particular groups. These slight differences can be caused by different plant material origins tested and different extraction and testing procedures utilized in experiments, but the main classes of identified compounds remain the same with the great advantage of phenolic compounds [18]. Generally, the metabolite profile of *Chamomilla recutita* is rich in compounds of proven pharmacological significance. Among the constituents of the tested sample, polyphenols were recognized as the most significant group of metabolites. In addition to their whitening properties, polyphenols from the flavonoid and phenolic acid groups have been shown to exhibit strong antioxidant potential. Their chemical structure enables them to efficiently scavenge free radicals by accepting and neutralizing them [19]. Due to these properties, polyphenols are often incorporated into various applications, including modern cosmetic formulations, as they exhibit a rejuvenating and anti-ageing effect on the skin, which complements the whitening action of a cosmetic. Several types of polyphenols are most abundant in the tested extracts. First are the simple organic acids, e.g., quinic, malic, and citric acids, as well as depsides, e.g., chlorogenic and ferulic acids. Additionally, compounds such as esculetin, methyl-umbelliferone, and dihydroxy-coumarin are also presented. Based on the scientific literature and our research, it can be concluded that chamomile is a rich source of various natural compounds and is worth further investigation.

### 2.3. Determination of Whitening Properties of Chamomile In Vivo in Zebrafish Model

*Danio rerio* can be an efficient model for analyzing whitening properties. Assays performed with zebrafish larvae enable the validation of results in vitro methods. According to the literature, zebrafish have melanophores located along the body, especially along the pigmentation bands. When tyrosinase is inhibited, the number of melanin-producing cells decreases, and the melanin pigment becomes lighter in color, resulting in a lighter pattern on the fish’s body. In Zebrafish larvae, loss of pigmentation can be seen in the caudal fin and along the dark stripes running down the sides of the body. Some melanogenesis inhibitors may affect pigmentation in the scalp area, especially if they affect neuroectodermal melanophore pathways, also the outermost layers of the skin, where melanophores are visible, may show gradual whitening [20,21,22].

**Figure 3:**
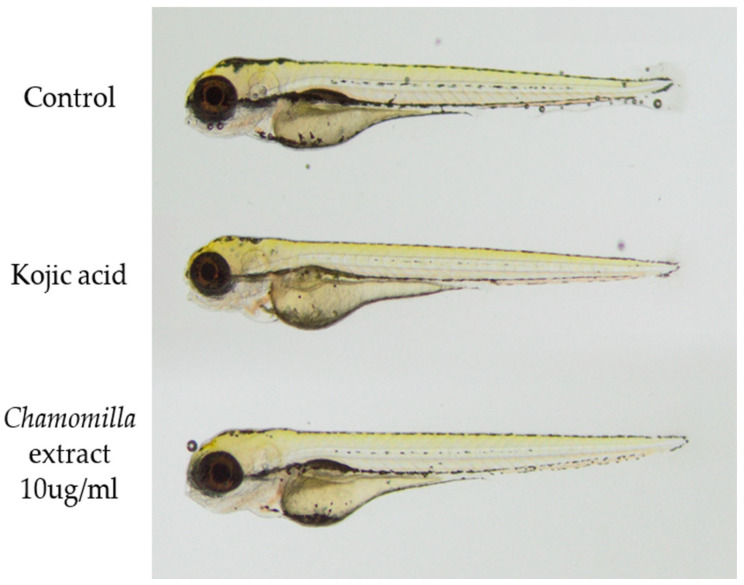
Visualization of zebrafish larvae in this study on the whitening properties of chamomile extract. The representative fish represent the negative control group, the positive control group (kojic acid), and the selected concentration of chamomile extract (10 μg/mL).

Based on these capabilities, we conducted studies using a water/ethanol extract from chamomile to determine if the suspected whitening action observed in the in vitro assay was effective enough and could function in a living organism. For this purpose, different concentrations of 50% EtOH extract from *M. recutita* flowers were screened for their impact on the pigmentation of *Danio rerio* larvae. Animals treated with chamomile extract exhibited a dose-dependent reduction in pigmentation. Among the tested groups, the most significant depigmentation was observed in animals treated with chamomile extract at a concentration of 10 uL/mL. The results confirmed the tyrosinase inhibition observed in vitro, which is presented in Figure 3. This study showed a clearly brightened pattern on the fish’s body, as visualized below, that resembled the color of fish from the kojic acid group. Moreover, in zebrafish larvae, loss of pigmentation was seen in the fluke and along the dark stripes running along the sides of the body, proving the whitening potential of the 50% EtOH extract from chamomile flowers.

There are only a few studies on the whitening properties of plant extracts or natural products that have been performed with a zebrafish model. For example, the natural compound T1, isolated from the plant *Gastrodia elata*, showed strong inhibitory activity against fungal tyrosinase and effectively reduced melanogenesis in zebrafish without any visible side effects [23]. In other studies performed on bark extract from a representative of the *Alnus* genus, namely, *Alnus cordata* (Betulaceae), the tested extract in in vitro studies showed a tyrosinase inhibitory potential with an IC_50_ value of 77.44 μg/mL and 39.58 μg/mL, calculated by L-DOPA auto-oxidation assay, proving that the constituents of the plant dose-dependently inhibited both the diphenolase activity of tyrosinase—the rate-limiting enzyme in melanin biosynthesis—and the spontaneous auto-oxidation of L-DOPA. In vivo studies on zebrafish embryos (72 h post-fertilization) demonstrated reduced body pigmentation without affecting the development and survival of the larvae with the most effective dose of 50 μg/mL, comparable to the effects of PTU treatment [24]. 

In relation to the results of the former study on *Alnus cordata* bark extract, the active concentration of chamomile flowers was 5 times lower and encouraged performing further studies on the obtained extract and, particularly, the identification of active components.

### 2.4. Bio-Chromatographic Studies Towards the Assignment of Single Molecules Interacting with Mushroom Tyrosinase Enzyme by HPLC-ESI-QTOF-MS/MS

The proved whitening potential of chamomile extract tested in a zebrafish model encouraged further studies on the identity of molecules responsible for whitening properties of the most active extract from chamomile.

**Figure 4:**
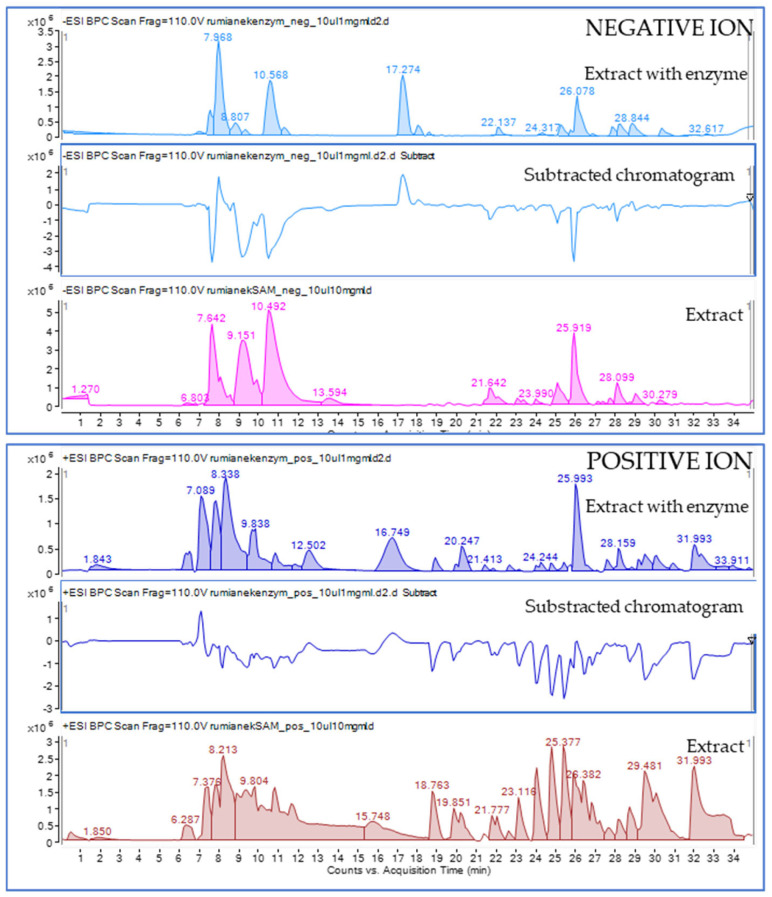
Mass chromatograms recorded in the negative and positive ion modes for the total water/ethanol extract from chamomile (extract), total extract mixed with tyrosinase enzyme (extract with enzyme), and the subtracted chromatogram obtained by the subtraction of the extract chromatogram from the extract with enzyme chromatogram (subtracted chromatogram).

To investigate potential interactions with tyrosinase, the chromatogram of the extract combined with the enzyme (extract with enzyme) was compared with the extract without the addition of an enzyme (extract), as shown in the Figure 4. As presented below, there are marked differences in the fingerprints of both analyzed samples. To achieve a better understanding of the outcomes of the extract–enzyme reaction, a subtracted chromatogram was prepared (subtracted chromatogram), which was generated by subtracting the extract chromatogram from the extract with the enzyme chromatogram, allowing for the identification of compounds interacting with the tyrosinase enzyme.

As shown in Figure 4, the subtracted chromatogram had a number of negative peaks, denoting decreases in intensity when reacting with the enzyme, which confirms the occurrence of interactions of the ligand–enzyme type, as previously elaborated by other researchers [25]. The following analysis of the *m*/*z* features corresponding to the negative peaks opened the door to a discussion on the potential active tyrosinase inhibitors present within the plant extract with a view to cosmetic applications. As a result, three chlorogenic acid derivatives represented the three negative peaks in the chromatogram registered in the negative ion mode, namely, at 23.068, 25.164, and 26.187 min. Large negative peaks that were observed at 7.6, 9.23, 10.5, 25.9, and 28.1 min resembled the *m*/*z* molecular features of 195.0523, 133.0148, 191.0158, and 355.1034, respectively, which were assigned to gluconic acid, malic acid, citric acid, and feruloyl hexose (two isomers at 25.9 and 28.1 min).

Additionally, a visible negative peak in the subtracted chromatogram of the negative ion mode was observed at 21.6 min. The *m*/*z* value of 315.0735 that was assigned to this signal corresponded with the MS/MS spectra of protocatechuoyl-glucose (see Table 2). The leading negative peak in negative mode that is present at 26.06 min in the subtracted chromatogram and the peak at 28.4 min in the same chromatogram come from the feruloyl-glucose isomers.

These results are in accordance with previous studies that state that organic acids show whitening properties on the skin. Results from Akiko Usuki’s research show that glycolic and lactic acid suppress melanin formation by directly inhibiting tyrosinase activity [26]. In addition, citric acid in studies on tyrosinase inhibition was listed as a whitening agent [27].

Among the tentatively identified molecules, phenolic acids also have an important place. Ferulic acid is a phenolic compound with strong antioxidant properties. Researchers proved its inhibitory potential against the tyrosinase enzyme, which led to the lightening of skin discolorations [28,29,30].

Moreover, numerous scientific papers list chlorogenic acid as a tyrosinase inhibitor [28,29]. Research indicates that it effectively binds with tyrosinase based on molecular docking simulations that reveal its interactions with specific amino acid residues of the enzyme. This binding suggests a potential mechanism through which chlorogenic acid or its derivatives could modulate tyrosinase activity and, consequently, melanin production [31,32,33]. Organic acids, phenolic acids, and their metabolites can act as tyrosinase inhibitors by binding to its active sites or other regions of the enzyme. The main chemical groups responsible for interaction with tyrosinase are hydroxyl groups (-OH), e.g., chlorogenic acid; carboxyl groups (-COOH), e.g., ferulic acid; and methoxy groups (-OCH_3_), e.g., feruloyl-glucose.

The characteristics of protocatechuoyl-glucose and feruloyl-glucose in terms of their tyrosinase inhibition have not been elaborated so far. However, their structural similarity to known TYR inhibitors (protocatechuic acid, ferulic acid) suggests potential inhibitory effects, warranting further investigation [34,35].

Collectively, these findings suggest that organic and phenolic acids and their metabolites, as simple structures, may serve as effective agents in modulating melanin synthesis through their interactions with tyrosinase, offering potential applications in cosmetic and therapeutic contexts targeting hyperpigmentation disorders.

**Figure 5:**
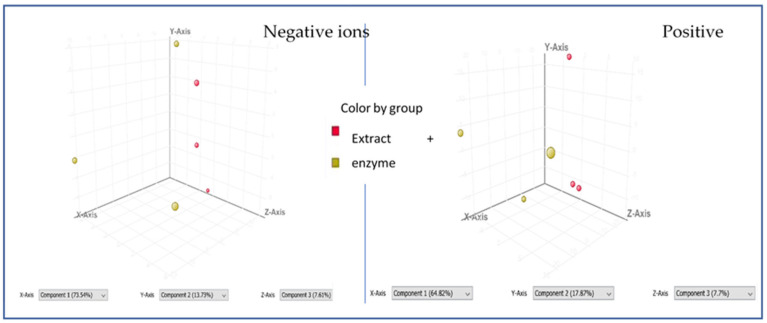
The results of the PCA analysis (*n* = 3) of the obtained injections of chamomile 50% EtOH extract in both positive and negative ion mode—alone and in combination with the mushroom tyrosinase.

The chemometric comparative analysis of the extract injections alone and after the treatment with the enzyme showed several *m*/*z* features that were differentiating the compared samples. As presented in Figure 5 below, two separated groups were formed from the extract injections and the extract–enzyme injections during a principal component analysis. PCA was performed on three separate injections and was calculated with *p* < 0.05 on 715 and 802 molecular features in negative and positive ion mode, respectively.

The performed study listed differentiating *m*/*z* features that were later analyzed by the authors of this work. They are presented in Table 3 below. Among a dozen different *m*/*z* features that at this point still remain not annotated, some were tentatively identified in our study while analyzing their fragmentation pattern.

Quinic acid (*m*/*z* of 191.0548), isopropylmalic acid (*m*/*z* of 175.0606), sinapic acid (*m*/*z* of 223.0612), coumaroyl-tartaric acid (281.0647), or caffeoylmalic acid (*m*/*z* of 295.0478) were present on the list of the differentiating molecular features. Also, the neutral mass of 508.1217 Da that was visible in the list of differentiating signals could be represented by the syringetin glucoside with *m*/*z* of 507.1144 that was traced in the extract; however, no MS/MS spectrum was recorded for this ion that could confirm its structure with a higher precision.

All the aforementioned compounds traced in the mass spectrometric analysis were previously reported to influence the activity of the tyrosinase enzyme. Among them, quinic acid was concerned as a potentially active tyrosinase inhibitor [36]. Next, chlorogenic acid was found to be the leading component of a whitening extract obtained from *Hypericum clycinum*. Also, malic acid, caffeic acid, and syringic acid and their derivatives were proved to show whitening properties in the formerly published literature. A study by Lin Gou and showed that malic acid can be an effective and safe tyrosinase inhibitor and suggested that the use of it may be useful in dermatological diseases [37].

In the next study, authors demonstrated the depigmenting mechanism of action of ethyl-2-acetamido-3-(4-hydroxy-3,5-dimethoxybenzoylthio)propanoate (EABTO), a thioester of plant-derived syringic acid, with N-acetylcysteine ethyl ester (NACET) carrying an L-cysteine residue [38].

### 2.5. Evaluation of the Tyrosinase Inhibitory Potential of Single Compounds

As the whitening potential was indicated in a study on a mixture of compounds, the last task of this project was to confirm the assumed properties in an in vitro assay on single components. For this purpose, the solutions of selected standards of phenolic and organic acids, whose interactions with the tyrosinase enzyme were proved in the proposed bio-chromatographic study on *M. recutita* extract, were screened for their IC_50_ values. As a result, the IC_50_ values were determined. For protocatechuic acid, the IC_50_ was 24.67 µM; for quinic acid, 22.90 µM; for chlorogenic acid, 12.46 µM; for ferulic acid, 0.484 µM; for caffeic acid, 5.53 µM; and for citric acid, 24.18 µM. Kojic acid was shown to have an IC_50_ of 0.22 µM under the described conditions. As presented, all tested standards demonstrated tyrosinase inhibitory potential, with ferulic acid being the most active inhibitor among the tested components, which is in accordance with previous studies [39].

**Figure 6:**
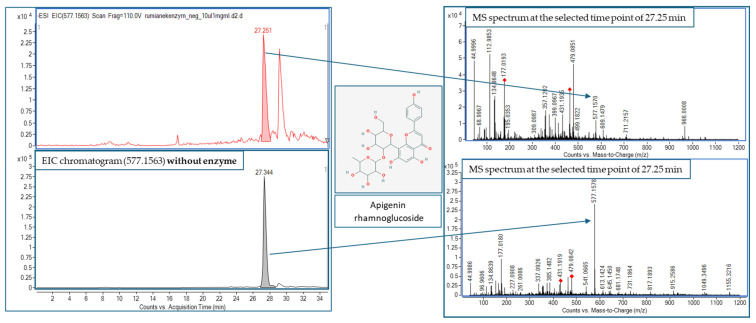
The analysis of apigenin rhamnoglucoside behavior upon the influence of tyrosinase in the recorded mass chromatograms—with and without the addition of enzyme (EIC—extracted ion chromatogram).

Also, we observed a marked decrease in the peak height in the major flavonoids glycosides that were present in the sample in a higher concentration, which confirms their ability to interact with the enzyme. This was the case of, e.g., apigenin rhamnoglucoside whose behavior is presented in the Figure 6, kaempferol malonylglucoside, or kaempferol arabinoside. Figure 6 clearly shows the decrease in the *m*/*z* of 577.1563 in the chromatogram obtained after the reaction with an enzyme and explains the concept of this work. A reduced signal intensity was due to the interaction with tyrosinase.

Interestingly, despite the wide representation of flavonoids, the aglycons that were traced in the tested extract of *M. recutita*, like apigenin or luteolin, did not show inhibitory properties in this assay. This is certainly due to their minute content in the sample and, possibly, the matrix effect, as they were coeluting with other metabolites at the similar retention time.

The results presented in this manuscript draw conclusions about the interactions between several phenolic and organic acids and the tyrosinase enzyme. The introduced approach constitutes a modification of the protocols previously described in the scientific literature and is called ligand fishing. These assays involve three steps: incubation of the biomolecule with potential ligands, washing (which was equal to filtering of our samples and removing the complexes with an increased molecular weight coming from the formed ligand–enzyme connections), and elution, which—in the case of this study—included the injection of the reagent mixture with the ligand–enzyme complexes removed for subsequent characterization [7]. A similar approach was described by Wang et al. [25], who searched for tyrosinase inhibitors in *Dryopteris crassirhizoma* extracts. In their study, the extracts and the fractions were mixed with tyrosinase enzyme, and the ultrafiltrated liquid was later assessed for changes in its fingerprint by HPLC-MS instrumentation. Similarly, the recognized tyrosinase inhibitors were observed as peaks of decreased area in the chromatograms compared with the control injection without an enzyme. This approach works well in plant extract samples.

Another study exploring potential tyrosinase inhibitors in *Vitis amurensis* employed the HPLC-MS method. The researchers fractionated the extract immediately after column separation, collecting the eluate in 96-well plates that were later subjected to spectrophotometric assays to evaluate their potential [40]. As a result, the tyrosinase inhibitory properties of Ɛ-viniferin and vitisin B were identified.

These two examples of research represent two approaches to performing affinity chromatography: pre-column derivatization, where components undergo enzymatic reactions before the supernatant is analyzed for changes, and post-column derivatization, where components are separated by HPLC and subsequently labelled. As noted by other authors, a challenge when analyzing complex samples in post-column reactions is the tailing of active molecules, which manifests as band broadening due to additional reagent volumes in the post-column reactors. This can obscure the activity of less prominent signals and saturate the overall signal. In contrast, pre-column reactions, as chosen in this study, offer high sensitivity and clarity in the obtained results, providing a better chance to observe the occurring interactions.

An evident advantage of pre-column reactions is the ability to easily manipulate extraction, chromatographic properties, and purification at this stage. This is particularly important in matrices rich in similarly polar components that may co-elute in the chromatographic column. However, a limitation of this approach is the potential formation of degradation products, which may lead to additional peaks in the chromatograms. Therefore, analyses should be performed as quickly as possible, under thermostated conditions, and the reactions should be halted promptly and centrifuged to minimize interference from potential precipitates. Certainly, the methodology may not fully resolve all formed protein–phenolic complexes. Future work involving size-exclusion methods or orthogonal separation techniques may help address this challenge.

Among the limitations of both approaches, issues with distinguishing between reversible enzyme inhibition and physical interaction/precipitation should be considered [41]. For the moment, it is difficult to differentiate the types of interactions between the components and the enzyme especially as the tyrosinase inhibition occurs in a competitive manner. Also, according to several authors, quercetin and kaempferol are rather substrates of the tyrosinase enzyme than inhibitors [42] and the studies on the elaboration of the interaction character between a compound and the enzyme would be of high value. However, with the usage of HPLC or standard spectrophotometric assays, conclusions of this nature cannot be drawn unequivocally.

Another important aspect is that both HPLC-based studies and spectrophotometric assays can deliver false-positive or false-negative results due to interactions in a complex sample that can be caused by matrix effects. This was also the case in our studies, where we noticed matrix effects hindering the activity of apigenin or luteolin that were co-eluting with other compounds at the same retention time. To solve this problem, the analysis of less complex samples is advised. Initial fractionation of the extracts, or better separation of metabolites on the chromatographic column, could assign a higher number of metabolites and better evaluate the sample–enzyme interactions of the minor constituents.

Still, the methodology employing mass spectrometry as a detection technique does not require using derivatizing agents, in contrast to HPLC, where post-column derivatization is often necessary. Lack of additional reagents certainly gives more clear spectra, reduced matrix effects, and lower limits of detection. It can be seen that recent instrumental advancements in online ligand fishing have introduced more complex setups that allow for simultaneous affinity assays and chromatographic separation. However, the implementation of these technologies necessitates specialized and costly equipment.

To sum up, the used approach—the analysis of a subtracted chromatogram from enzyme-treated and non-treated extract solution, as well as the application of chemometric tools for the direct comparison of the *m*/*z* signal identity—provided much evidence on the differentiating signals and led to a tentative assignment of various compounds with tyrosinase inhibitory action. This particular approach may be helpful in identifying potential active principles present in a mixture without the need for their time- and resource-consuming isolation. Also, it may help to identify in a mixture of compounds the molecules with an unknown so far activity for further biological assessment. The described methodology is repetitive, resource-saving, and—what is most important—universal. It may be used to assess different types of enzymatic reactions not only with tyrosinase, but with a view to application with other enzymes. Despite these advantages, the approach may be used for initial screening, best on fractionated extracts, and should be followed by bioactivity-guided isolation studies that could confirm the accuracy of the bio-chromatographic screening in further assays on purified components.

## 3. Materials and Methods

### 3.1. Plant Material and Extraction

Dried plant material from the selected species of herbs growing in the Lubelskie region was purchased from the Krautex company (Łopiennik Górny, Poland) and comminuted prior to the extraction procedure. Among the tested plants, the herb of *Chamomilla recutita* (Series number: 0360509233), the flower of *Calendula officinalis* (Series number: 1050509233), the rhizome of *Taraxacum officinale* (Series number: 0371309233), the fruit of *Rosa canina* (Series number: 1063008233), the flower of *Sambucus nigra* (Series number: 0202609233), the fruit of *Crataegus* *monogyna* (Series number: 0211109233), the herb of *Urtica dioica* (Series number: 0352209233), and the herb of *Equisetum arvense* (Series number: 0072508233) were used for the extraction and evaluation of tyrosinase inhibition.

In the extraction process, five hundred milligrams of the powdered plant material were suspended in 1.5 mL of the extracting solvent in a 2 mL Eppendorf vial. A series of extracts using ethanol, water, and ethanol/water (1:1 *v*/*v*) mixture were prepared from separate portions of plant material using ultrasound-assisted extraction technique for 15 min at room temperature (ultrasonic bath, Sonic-3, Polsonic, Warsaw, Poland, operating at a high frequency of 35 kHz). The extracts were later centrifuged, and the supernatant was filtered through a 0.1 µm syringe nylon filter into a weighted vial and evaporated to dryness using the Concentrator Plus system (Eppendorf, Warszawa, Poland) at a temperature of 45 °C. The samples were weighted and stored at 4 °C until analysis, but not longer than for a period of two weeks.

### 3.2. In Vitro Biological Activity Determination Towards the Tyrosinase Inhibitory Potential

The inhibitory properties of the extracts against tyrosinase were evaluated using in an in vitro assay with the mushroom tyrosinase enzyme and L-DOPA as a substrate (Sigma Aldrich, St. Louis, MO, USA). The assay was performed based on the method by Uchida and colleagues [43]. Briefly, 100 µL of 100 mM phosphate buffer (pH 6.8) and 20 µL of the diluted extracts of studied plants (1 mg/mL solution in DMSO) were pipetted into a 96 well microwell. Afterwards, 20 μL of the mushroom tyrosinase (500 U/mL) dissolved in phosphate buffer (pH 6.8) was added to each microwell and the mixture was pre-incubated for 10 min at room temperature. After incubation, 80 µL of 8 mM L-DOPA was added and the reaction was initiated and sustained at 25 °C. After 20 min, the absorbance was measured at 450 nm using a microplate reader Promega GloMax Explorer GM 3510 (Madison, MO, USA). The control sample (with 100% tyrosinase activity) contained the appropriate volume of the solvent (100 µL buffer plus 20 µL DMSO) instead of the extract. In both assays, the dopachrome formation was measured spectrophotometrically at λ = 450 nm, using a Promega microplate reader. The obtained values were corrected by the absorbance value of the background that was measured for the extracts without the mushroom tyrosinase and L-DOPA. Each sample was analyzed in three independent repetitions. Kojic acid dissolved in DMSO at a concentration of 1 mg/mL was used as a known TYR inhibitor—as a positive control.

The same protocol was utilized to determine the IC_50_ values of the individual components—protocatechuic, quinic, chlorogenic, kojic, ferulic, caffeic, and citric acids—that were identified by the chromatographic studies as interacting with the tyrosinase enzyme. The assay was conducted using reference compound solutions with a purity exceeding 95%, purchased from Sigma Aldrich (St. Louis, MO, USA). For the determination of the IC_50_ values and the inhibitory potential, the test solutions encompassed a concentration range of 0.015 to 5.0 mg/mL.

### 3.3. Compositional Studies of the Most Active Extract by HPLC-ESI-QTOF-MS/MS Approach

The chromatographic and bio-chromatographic studies were performed in an HPLC-ESI-QTOF-MS/MS platform produced by Agilent Technologies (Santa Clara, CA, USA). The system consisted of a 1200 Series HPLC chromatograph, which included a degasser, autosampler, column thermostat, DAD detector, and a mass spectrometer (G6530B) with a dual AJS ESI ionization source. Additionally, an isocratic pump was used for delivering the reference ion mixture (Agilent Technologies). The extracts used for the chromatographic analysis were re-dissolved in the respective solvents to obtain a concentration of 10 mg/mL. They were centrifuged, and the resulting supernatant was filtered through a nylon syringe filter with a pore size of 0.1 µm directly into autosampler vials. The chromatographic separation used for chamomile 50% EtOH extract was achieved in the gradient of water with 0.1% formic acid with acetonitrile with an addition of 0.1% formic acid (B): 0 min—1% B; 10 min—20% B; 20 min—40% B; 22–26 min—95% B; 27–35 min—1% B; separation was performed on a C18 Zorbax Eclipse Plus column with pore size of 3.5 µm and the dimensions of 150 mm × 2.1 mm.

Mass spectra were obtained after injecting 10 µL of the sample into the freshly calibrated instrument using two ionization modes, with three repetitions for each mode. The MS settings were as follows: gas temperature at 250 °C, shield gas temperature at 300 °C, gas flow at 12 L/min, nebulizer pressure of 35 psig, skimmer voltage at 65 V, capillary voltage at 3000 V, nozzle voltage at 1000 V, fragmentor voltage at 110 V, and an *m*/*z* range of 40–1200 Da. Collision energies of 10 and 20 V were applied. The two most intense *m*/*z* signals in each scan were fragmented and then excluded for 0.2 min in the subsequent scans. Data acquisition and analysis were managed using Mass Hunter Workstation software (version B.10.00, Agilent Technologies). Sample processing, which involved identifying molecular features according to spectral libraries (Metlin: https://metlin.scripps.edu/landing_page.php?pgcontent=mainPage, NIST: https://webbook.nist.gov/chemistry/, accessed on 20 January 2025), was performed with the Mass Profiler Professional Program (version 15.1, Agilent Technologies).

### 3.4. Zebrafish Assay for the Determination of Whitening Properties of Chamomile 50% EtOH Extract

#### 3.4.1. Animals and Ethical Approval

Zebrafish embryos of the AB strain were obtained from the zebrafish facility at the Experimental Medicine Centre of the Medical University of Lublin, Poland. They were reared under standard laboratory conditions at 28 °C. Larvae up to 4 days post-fertilization (dpf) were used for the experiments. Ethical approval was not required for the studies involving embryos and larval zebrafish up to 120 h post-fertilization (hpf). A tricaine solution was used for the euthanasia of the tested larvae of zebrafish.

#### 3.4.2. Determination of Whitening Properties of Chamomile In Vivo in Zebrafish Model

To prove the whitening potential of polyphenol-rich extract of *Chamomilla recutita* in zebrafish, the 50% EtOH extract was used. Melanin pigments can be observed on the surface of zebrafish, allowing for simple observation of the pigmentation process. For monitoring the melanogenic inhibitory activity, 0 hpf embryos were selected and put in 300 µL embryo medium E3 (1.5 mM HEPES, pH 7.6, 17.4 mM NaCl, 0.21 mM KCl, 0.12 mM MgSO_4_, and 0.18 mM Ca(NO_3_)_2_) in 48-well plates. Next, the embryos were treated with *Chamomilla recutita* extract at various concentrations, i.e., 1000 µL/mL, 750 µL/mL, 500 µL/mL, 250 µL/mL, 100 µL/mL, 50 µL/mL, and 10 µL/mL. The tested extract was dissolved in DMSO, which was consistently used as a vehicle control at a final concentration of up to 0.1% (*v*/*v*). In all experiments, kojic acid (2 mM), a well-known TYR inhibitor, served as the standard positive control [44,45]. The testing period ranged from 24 to 48 h, and pigmentation effects were monitored using a stereoscopic microscope. Larvae at 96 hpf were mounted in methylcellulose and photographed (Olympus SZ61, Olympus, Tokyo, Japan). The effect on melanocyte development after 96 hpf is shown in Figure 3.

### 3.5. Identification of Individual Components Within the 50% EtOH Extract of Chamomilla recutita Extract Exhibiting Tyrosinase Inhibitory Potential in HPLC-MS Approach

For the identification of single components responsible for interactions with tyrosinase enzymes, a bio-chromatographic assay was performed. For this purpose, two 0.5 mL portions of 1 mg/mL 50% EtOH extract of chamomile dissolved in water each were transferred to an Eppendorf vial of 2 mL. The sample coded as sample A was mixed with 0.5 mL of carbonate buffer prepared by dissolving 120 mg Na_2_CO_3_ and 12.5 mg NaHCO_3_ in 1000 mL of water and later diluted 40 times, whereas sample B was mixed with 0.5 mL of tyrosinase enzyme (500 U/mL) in the same carbonate buffer and diluted 40 times as sample A was.

Both samples A and B were filtered through nylon syringe filters (0.1 µm pore size) into autosampler vials and analyzed using an HPLC-ESI-QTOF-MS/MS instrument. The test was performed in triplicate. During the analysis of recorded data, the obtained chromatograms were subtracted from each other in the Mass Hunter Workstation Program to highlight the changes visible under the influence of the enzyme, and some of the potentially active components were tentatively identified thanks to high-resolution mass measurement, recorded MS/MS fragmentation patterns, and comparison with open mass databases (Metlin, HMDB, Mass Bank). Both injections—with and without the enzyme—were also compared using the Mass Profiler Professional Program (Agilent Technologies, Santa Clara, CA, USA). For this purpose, molecular feature extraction was carried out using the Profinder program v. 10.0.02 (Agilent Technologies, Santa Clara, CA, USA) to obtain a list of *m*/*z* values, retention times, and peak areas from the recorded injections, which were exported in profinder archive and detailed CSV formats for subsequent chemometric comparison. The data were filtered based on criteria including a mass tolerance of 10 ppm, retention time variation of 0.3 min, and a minimum peak area of 1% of the highest signal. The chemometric analysis performed directly within the program yielded 802 entities in positive ion mode and 715 in negative ion mode (*p* < 0.05), in a one-way ANOVA test with asymptotic *p*-value computation and Benjamini–Hochberg multiple testing correction. The identification of differentiating signals was achieved at *p* < 0.001, generating a list of high-resolution mass measurements for comparison with MS libraries such as the Metlin collection (which includes metabolites, lipids, proteins, and small molecules), as well as the relevant scientific literature.

## 4. Conclusions

The aim of this study was to present an approach for the identification of individual molecules present in a mixture that exhibit tyrosinase inhibitory potential, utilizing affinity chromatography. Among the screened plants from the Lubelskie region and their extracts at varying polarity, the 50% EtOH extract of chamomile was selected as the most effective in a spectrophotometric assay with mushroom tyrosinase. The selected extract was found to contain a diverse range of polyphenols and organic acids, as confirmed by HPLC-ESI-QTOF-MS/MS analysis, making it an interesting matrix for further bioactivity studies. The application of a bio-chromatographic approach to chamomile extract, combining chromatographic techniques with enzyme-based assays, enabled the tentative identification of individual components in the extract that interacted with tyrosinase. This was accomplished by injecting two samples, one with the enzyme and one without, into a chromatographic column, followed by HPLC-ESI-QTOF-MS/MS analysis. Among the identified components influenced by the presence of the enzyme were several organic acids (gluconic, malic, citric, and quinic acids), and phenolic acids and their glucosides (the derivatives of syringic, ferulic, caffeoylquinic, and coumaric acids). The aforementioned components had been previously demonstrated to possess whitening properties, thereby affirming the validity of the analysis and highlighting the potential applications of mass spectrometry coupled with liquid chromatography in analyzing a wide range of plant extracts for their enzyme inhibitory properties.

The described methodology is straightforward, reproducible, and can be extended to other enzymes and different plant extracts. In the case of chamomile, the performed study confirmed its whitening properties and—along with a detailed analysis of its qualitative fingerprint—led to the identification of specific molecules responsible for this effect. The analyzed extract was found to inhibit the synthesis of melanin in zebrafish larvae, giving confirmation to the outcomes of the in vitro assays.

The active metabolites identified may serve as effective ingredients in products aimed at reducing hyperpigmentation and promoting skin health. Future studies on chamomile should focus on optimizing extraction methods to sustain the highest possible recovery from plant matrix and enable further in vivo studies to validate these findings.

Certainly, the proposed methodology has certain limitations. However, the introduction of HPLC chromatographs significantly reduces the need for consumables required to fractionate mixtures and enables highly selective and sensitive studies of ligand–substrate interactions. Overall, these methodologies enhance the efficiency and reliability of bioactive compound discovery.

## Figures and Tables

**Table 1:** The results of the tyrosinase inhibitory potential in mushroom tyrosinase for the selected plant species from the Lubelskie region presented as mean values ± SD (*n* = 3) (remaining tyrosinase activity—the measured activity of the enzyme; tyrosinase inhibition—the percentage of inhibited enzyme activity, which was calculated by subtracting the remaining tyrosinase activity from 100, with results expressed as values greater than 0).

Species Name	Type of Extract	Remaining Tyrosinase Activity [%]	Tyrosinase Inhibition [%]
Chamomile herb *Matricaria chamomilla*	96% EtOH 50% EtOH H_2_O	128 ± 4.60 24.2 ± 7.18 60.6 ± 3.95	0 75.8 39.4
Marigold flower *Calendula officinalis*	96% EtOH 50% EtOH H_2_O	127 ± 26.1 58.4 ± 3.41 56.8 ± 1.43	0 41.6 43.2
Dandelion rhizome *Taraxacum officinale*	96% EtOH 50% EtOH H_2_O	62.9 ± 15.6 61.5 ± 3.35 72.3 ± 5.14	37.1 38.5 27.7
Rosehip fruit *Rosa canina*	96% EtOH 50% EtOH H_2_O	111 ± 23.5 184 ± 8.71 167 ± 20.3	0 0 0
Elderberry flower *Sambucus nigra*	96% EtOH 50% EtOH H_2_O	316 ± 17.4 200 ± 3.60 115 ± 3.03	0 0 0
Hawthorn fruit *Crataegus* *monogyna*	96% EtOH 50% EtOH H_2_O	180 ± 27.9 194 ± 9.41 156 ± 10.5	0 0 0
Nettle herb *Urtica dioica*	96% EtOH 50% EtOH H_2_O	224 ± 28.9 183 ± 1.41 114 ± 20.5	0 0 0
Horsetail herb *Equisetum arvense*	96% EtOH 50% EtOH H_2_O	259 ± 1.81 151 ± 11.5 276 ± 4.75	0 0 0
Kojic acid (1 mg/mL)	H_2_O	42.0	57.97

**Table 2:** The tentatively identified components of the chamomile extract recorded in the negative and positive ion modes by HPLC-ESI-QTOF-MS/MS analysis (Rt—retention time; DBE—number of double bonds and rings); the identification was based on the retention time analysis, fragmentation pattern, and the scientific literature data [15,16,17].

No	Ion	Rt [min]	Proposed Compound	Neutral Molecular Formula	Theoretical Mass	Experimental Mass	MS/MS Fragments	Error of Measurement [ppm]	DBE
1	[M−H]^−^	7.0514	Gluconic acid	C_6_H_12_O_7_	195.0510	195.0523	177.0404 129.0192 99.0091 75.0097	−6.5	1
2	[M−H]^−^	8.078	Quinic acid	C_7_H_12_O_6_	191.0561	191.0563	173.0555 127.0475 103.0099	−0.98	2
3	[M−H]^−^	9.587	Malic acid	C_4_H_6_O_5_	133.0142	133.0148	114.9936 89.0169 71.0086	−4.12	2
4	[M−H]^−^	10.459	Citric acid	C_6_H_8_O_7_	191.0197	191.0198	173.0064 129.0168 111.0068 87.0074	−0.38	3
5	[M−H]^−^	17.618	Baicalin	C_21_H_18_O_11_	445.0776	445.0788	-	−2.61	13
6	[M−H]^−^	21.609	Protocatechuoylglucose	C_13_H_16_O_9_	315.0722	315.0731	203.0012 165.0184 152.0111 108.0214	−2.99	6
7	[M−H]^−^	21.860	Glucogallic acid	C_13_H_16_O_10_	331.0671	331.0673	313.0577 168.0061 125.0238	−0.69	6
8	[M−H]^−^	22.917	Dihydroferulic acid 4-*O*-glucuronide;	C_16_H_20_O_10_	371.0984	371.0988	251.0550 209.0314 197.0468 191.0258 167.0335	−1.15	7
9	[M−H]^−^	23.068	Chlorogenic acid	C_16_H_18_O_9_	353.0872	353.0882	209.0299 191.0557 179.0349 161.0232 135.0444	−1.11	8
10	[M−H]^−^	23.4	Dihydroxybenzoic acid	C_7_H_6_O_4_	153.0193	153.0195	109.0292 81.0350	−1.09	5
11	[M−H]^−^	23.403	Protocatechuic acid	C_7_H_6_O_4_	153.0212	153.0198	109.0293 91.0189 81.0352	−3.4	5
12	[M−H]^−^	24.845	Isopropylmalic acid	C_7_H_12_O_5_	175.0612	175.0606	157.0456 131.0659 115.0352	3.39	2
13	[M−H]^−^	25.164	Neochlorogenic acid	C_16_H_18_O_9_	353.0872	353.0856	191.0529 179.0312 173.0426 161.0211	6.23	8
14	[M−H]^−^	25.248	Syringetin glucoside	C_23_H_24_O_13_	507.1144	507.1150	-	−1.15	12
15	[M−H]^−^	25.751	Feruloyl hexose	C_16_H_20_O_9_	355.1034	355.1040	193.0499 149.0594 134.0358	−1.53	7
16	[M−H]^−^	25.919	Syringin	C_17_H_24_O_9_	371.1348	371.1353	209.0647 191.0683 179.0515 149.0459	−1.46	6
17	[M−H]^−^	26.003 and 28.434	Feruloyl-glucose isomers	C_16_H_20_O_9_	355.1029	355.1042	241.1059 193.0494 149.0598 134.0364	−2.09	7
18	[M−H]^−^	26.103	Ferulic acid	C_10_H_10_O_4_	193.0506	193.0513	149.0592 134.0363	−3.44	6
19	[M−H]^−^	26.1870	(Z)-chlorogenic acid	C_16_H_18_O_9_	353.0872	353.0875	191.0550 161.0235 149.0595 134.0360 119.0337	−4.78	8
20	[M−H]^−^	26.606	*p*-Coumaroyltartaric acid	C_13_H_14_O_7_	281.0667	281.0647	-	7.01	7
21	[M−H]^−^	27.009	7,8-Dihydroxycoumarin	C_9_H_6_O_4_	177.0193	177.0193	149.0202 133.0257 105.0311	0.18	7
22	[M−H]^−^	27.076	Esculetin	C_9_H_6_O_4_	177.0193	177.0202	149.0209 133.0251 121.0247 105.0310	−4.87	7
23	[M−H]^−^	27.260	Apigenin-7-*O*-rhamnoglucoside (Rhoifolin)	C_27_H_30_O_14_	577.1563	577.1568	413.0882 293.0461	−0.9	13
24	[M−H]^−^	27.361	Caffeic acid	C_9_H_8_O_4_	179.035	179.0357	163.0052 135.0444 93.0340	−3.99	6
25	[M−H]^−^	27.746	Luteolin-7-*O*-rutinoside	C_27_H_30_O_15_	593.1506	593.1534	285.0401	−3.71	13
26	[M−H]^−^	28.233	Caffeoylmalic acid	C_13_H_12_O_8_	295.0459	295.0478	179.0343 163.0365 133.0135 115.0026	−6.28	8
27	[M + H]^+^	28.308	4-Methylumbelliferone	C_10_H_8_O_3_	177.0546	177.0554	149.0590 133.0647 121.0651	−4.43	7
28	[M−H]^−^	28.669	Luteolin galactoside	C_21_H_20_O_11_	447.0933	447.0934	327.0514 285.0396 256.0364 151.0020	−0.26	12
29	[M−H]^−^	28.669	Kaempferol glucoside	C_21_H_20_O_11_	447.0934	447.0933	285.0396	0.26	12
30	[M−H]^−^	28.719	Patulitrin	C_22_H_22_O_13_	493.0990	493.0988	331.0447 316.0209 287.0193 181.0126	0.48	12
31	[M−H]^−^	28.736	Glucocaffeic acid	C_15_H_18_O_9_	341.0881	341.0891	281.0611 251.0487 179.0297 161.0204 135.0398	−3.78	7
32	[M−H]^−^	28.736	Hyperoside	C_21_H_20_O_12_	463.0891	463.0896	300.0274 271.0225 151.0012	−3.02	12
33	[M−H]^−^	29.189	Propylglutaric acid	C_8_H_14_O_4_	173.0819	173.0809	-	5.93	2
34	[M−H]^−^	29.574	Hydroxydecanoic acid	C_10_H_18_O_5_	217.1081	217.1076	199.0939 171.1004 155.1064 137.0957 127.1105	2.51	2
35	[M−H]^−^	29.776	Di-caffeoylquinic acid	C_25_H_24_O_12_	515.1195	515.1203	353.0854 335.0794 308.1009 191.0540 179.0340 173.0435	−1.55	14
36	[M−H]^−^	30.262	Isorhamnetin 3-*O*-glucoside	C_22_H_22_O_12_	477.1053	477.1038	315.0617 299.0130 285.0347 243.0349 161.0189 152.0057 108.0163	3.03	12
37	[M−H]^−^	30.396	Kaempferol 3-(6″-malonylglucoside)	C_24_H_22_O_14_	533.0950	533.0937	489.1002 285.0350 150.9987	2.47	14
38	[M−H]^−^	30.447	Sinapic acid	C_11_H_12_O_5_	223.0612	223.0604	179.0651 163.0362 133.0623	3.56	6
39	[M−H]^−^	30.530	Apigenin 8-C-glucoside	C_21_H_20_O_10_	431.0984	431.0964	268.0321	4.56	12
40	[M−H]^−^	30.933	Di-caffeoyl-quinic acid	C_25_H_24_O_12_	515.1205	515.1195	353.0854 191.0533 179.0325 173.0424 161.0240 135.0435	1.94	14
41	[M−H]^−^	31.117	Di-caffeoylquinic acid	C_25_H_24_O_12_	515.1195	515.1196	353.0880 323.0773 191.0549 179.0337 173.0441 161.0237	−0.19	14
42	[M−H]^−^	31.537	Kaempferol-3-*O*-alpha-L-arabinoside	C_20_H_18_O_10_	417.0827	417.0849	-	−5.21	12
43	[M−H]^−^	31.620	Apigenin	C_15_H_10_O_5_	269.0455	269.0462	-	−2.42	11
44	[M−H]^−^	32.62	Luteolin	C_15_H_10_O_6_	285.0405	285.0419	-	−5.03	11

**Table 3:** The list of *m*/*z* features differentiating the extract before and after the treatment with the tyrosinase enzyme.

Positive Ion Mode			Negative Ion Mode		
*m*/*z* of a Neutral Ion	Retention Time	Regulation	*m*/*z* of a Neutral Ion	Retention Time	Regulation
78.0148	9.2	up	176.0655	25.094	up
157.111	9.265	up	298.0699	24.3819	up
175.0047	10.244	up	224.0664	30.636	up
522.158	10.244	up	129.9671	7.013	up
80.011	9.185	up	296.056	28.121	up
312.0026	14.006	up	192.062	26.241	up
334.0302	10.23	up	282.0723	26.569	up
291.9572	10.272	up	280.056	7.798	up
369.9593	10.223	up	240.0636	25.76	up
260.1743	8.679	up	328.0932	19.036	up
541.7257	7.159	up	297.8905	7.027	up
457.7642	7.149	up	508.1229	25.266	up
376.0027	9.937	up	120.0432	7.8	up
335.838	6.951	up			
179.0642	21.53	up			
543.7241	7.1579	up			
109.0024	9.8	down			
257.2374	0.956	down			
241.9188	6.926	down			
259.9874	9.33	down			

## Data Availability

The scientific data obtained during this study are all present in the manuscript and Supplementary Materials.

## Supplementary Materials

The following supporting information can be downloaded at: https://www.mdpi.com/article/10.3390/molecules30091979/s1, Figure S1: Representation of different classes of compounds found to be active tyrosinase inhibitors with the analysis of their fragmentation pattern; Table S1: MS/MS spectra of the tentatively identified components of chamomile extract recorded at 10 or 20 eV CID energy.
