# Supplementary material for: Tyrosinase Inhibitors Among Flora of Lubelskie Region—Application of Bio-Chromatographic Approach and Zebrafish Model in Bioactivity Screening of Plant Material
Source: Molecules. 2025 Apr 29;30(9):1979. doi: 10.3390/molecules30091979 (PMC12073248; doi:10.3390/molecules30091979)

Suplementary file

Table S1. The MS/MS spectra of the tentatively identified components of chamomile extract recorded at 10 or 20 eV CID energy.

Name	MS/MS fragment
**Chlorogenic acid**	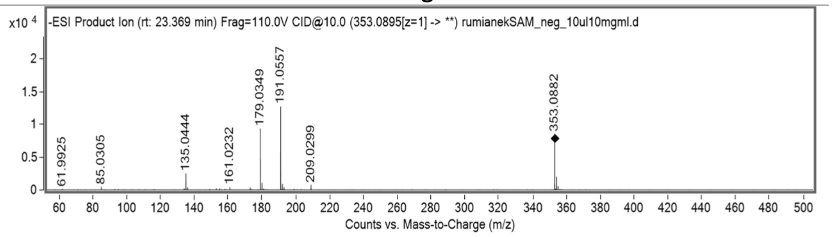
**Neochlorogenic acid**	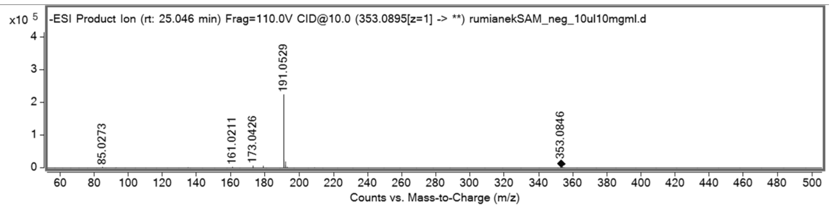
**(Z)-Chlorogenic acid**	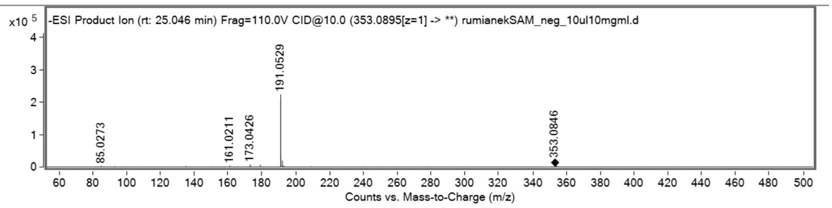
**Feruloyl-glucose isomers**	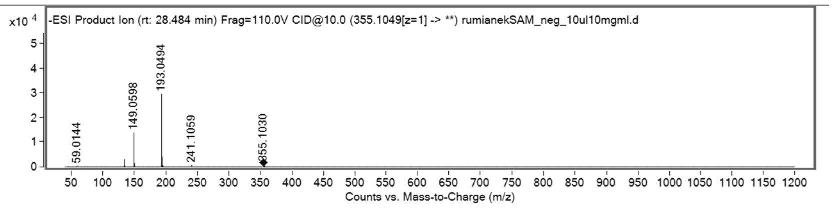
**Luteolin-7-*O*-rutinoside**	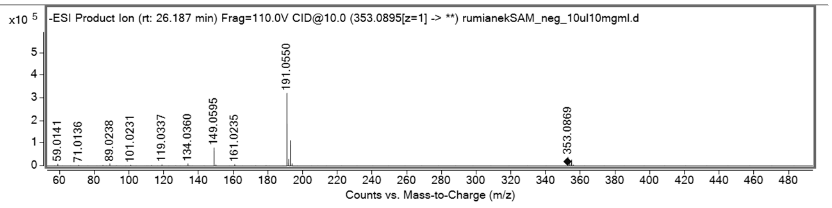

**1,4-Di-O-caffeoylquinic acid**	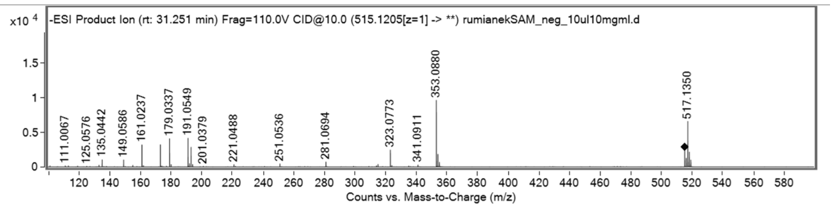
**Quinic acid**	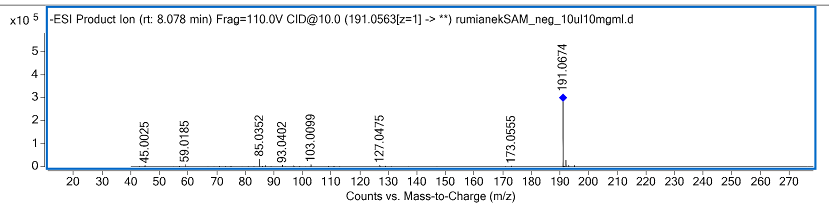
**Hyperoside**	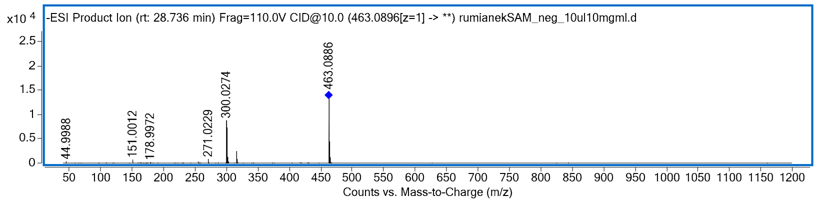
**Glucocaffeic acid**	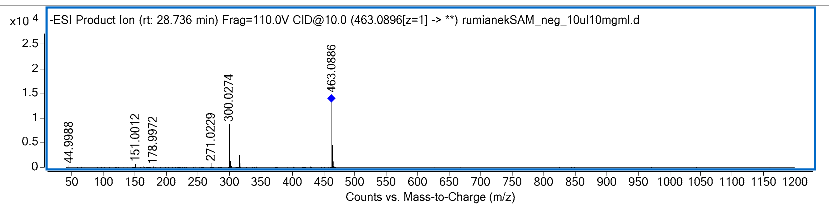
**Ferulic acid**	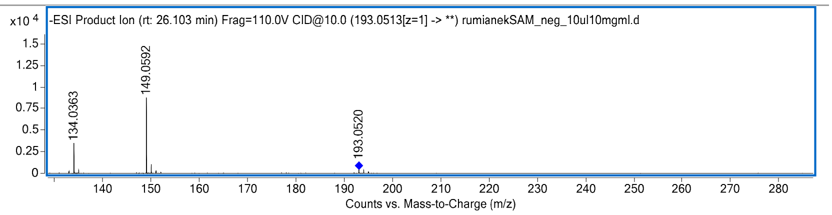

Esculetin

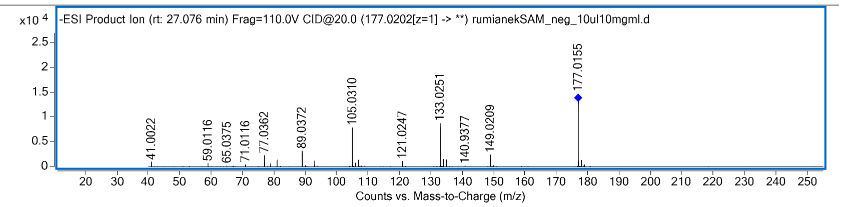

4-methylumbelliferone

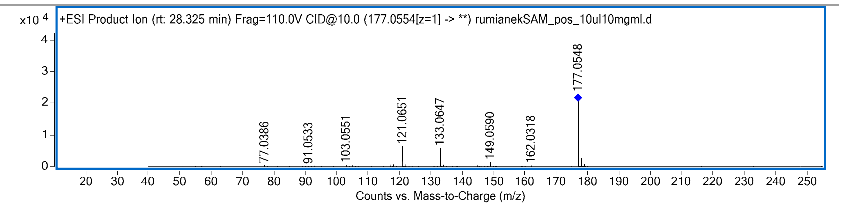

Syringin

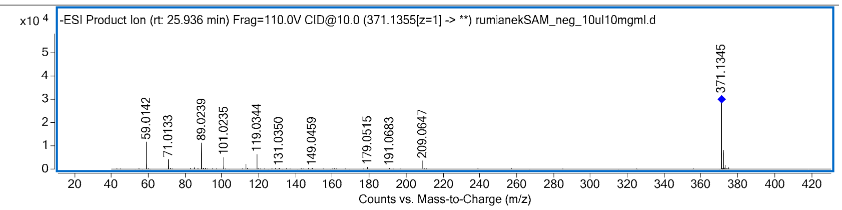

Dihydroxybenzoic acid

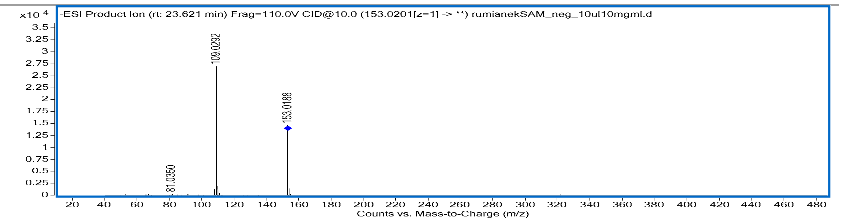

Glucogallic acid

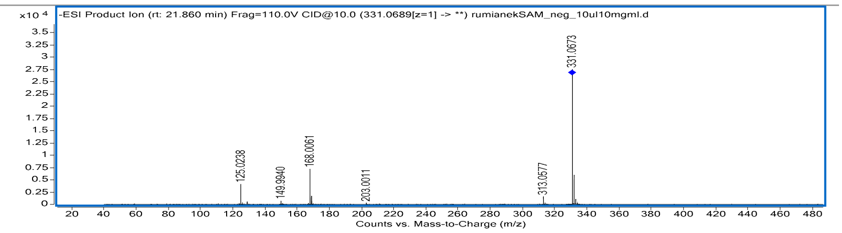

Dihydroferulic acid 4-O-glucuronide

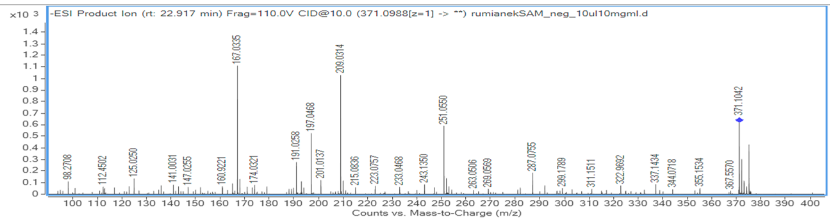

Luteolin galactoside

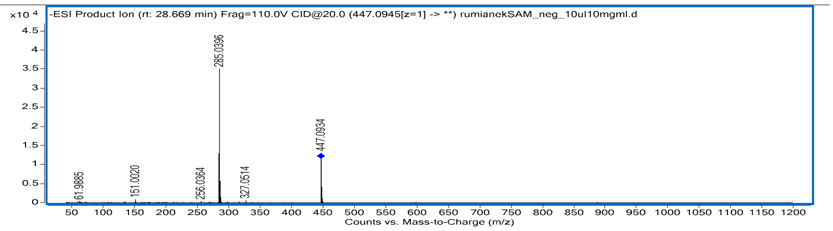

Hydroxydecanoic acid

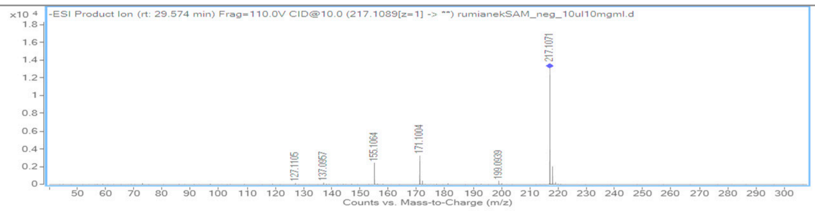

Protocatechuoylglucose

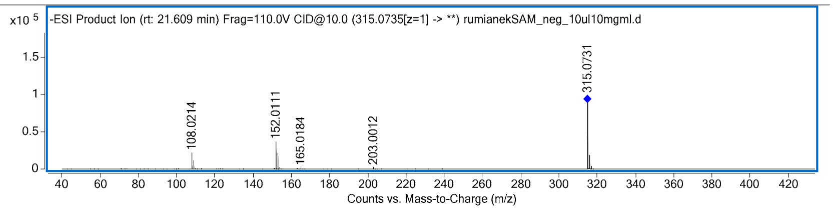

Caffeoylmalic acid

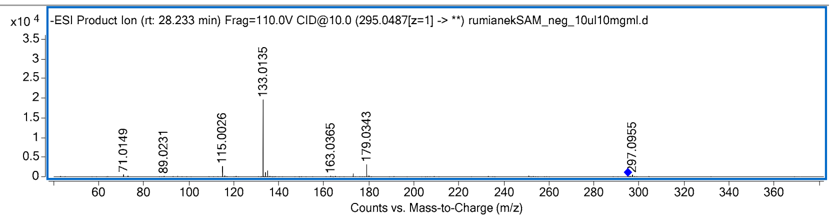

Isopropylmalic acid

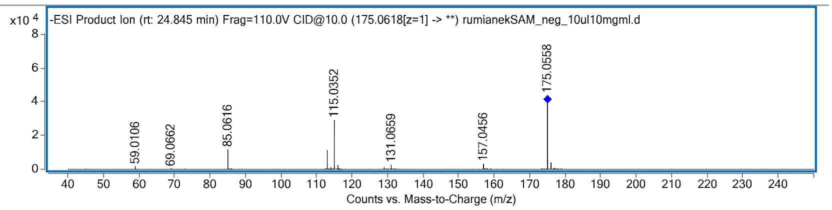

Sinapic acid

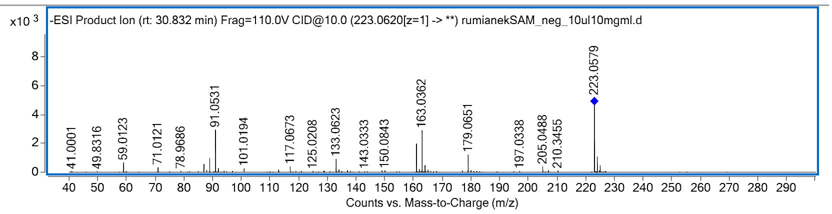

p-coumaroyltartaric acid

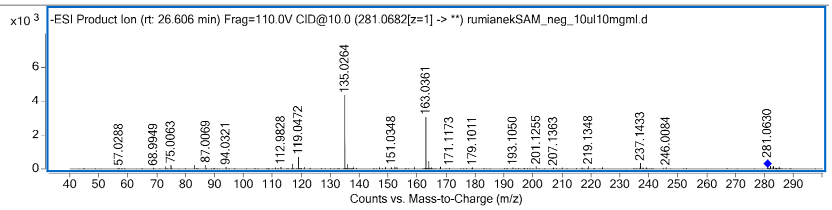

Caffeic acid

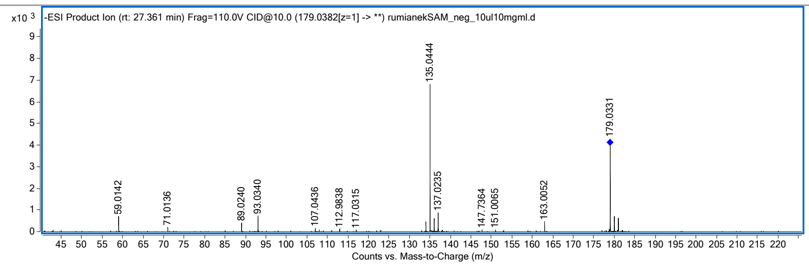

Protocatechuic acid

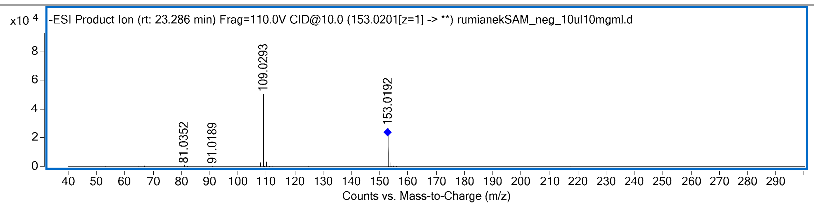

7,8-Dihydroxycoumarin

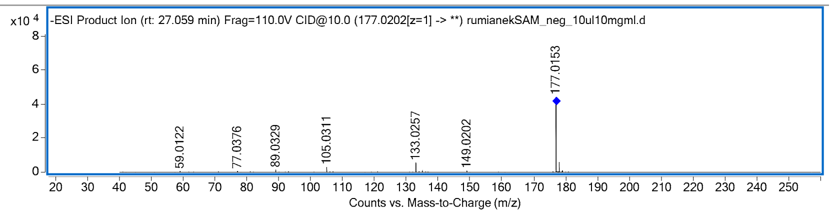

Apigenin-7-O-rhamnoglucoside (Rhoifolin)

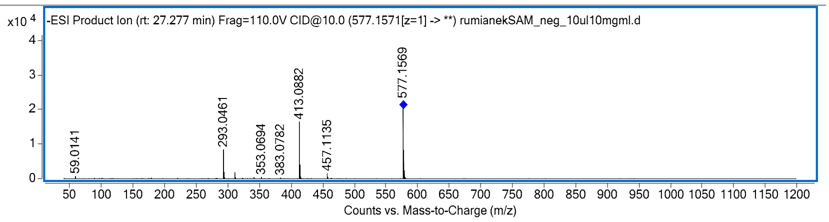

Apigenin 8-C-glucoside

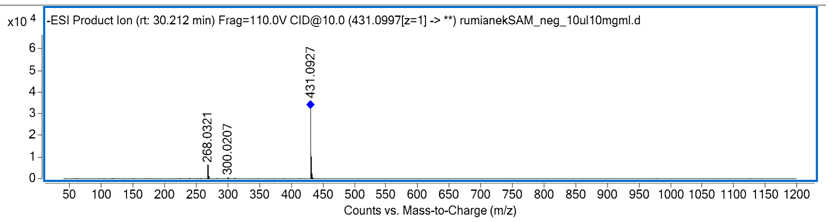

Citric acid

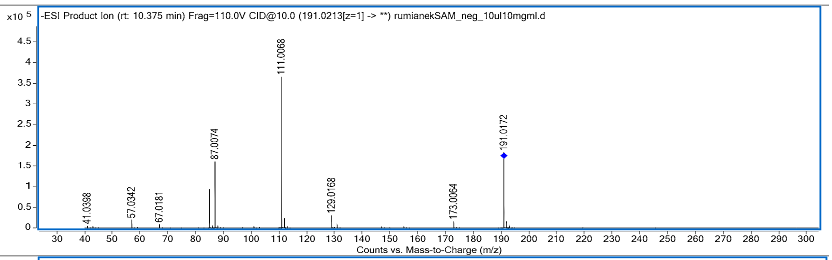

Malic acid

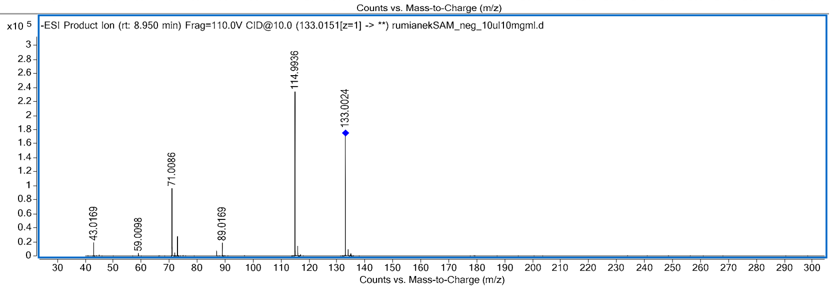

Gluconic acid

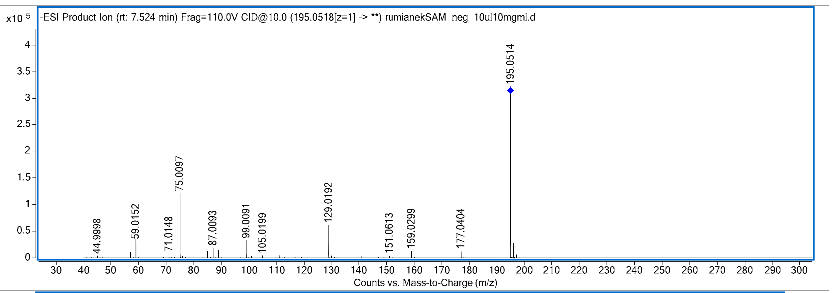

Feruoyl hexose

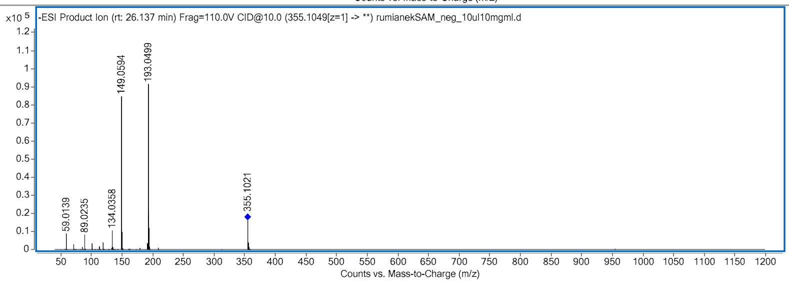

Figure S1. The representation of different classes of compounds found to be active tyrosinase inhibitors with the analysis of their fragmentation pattern

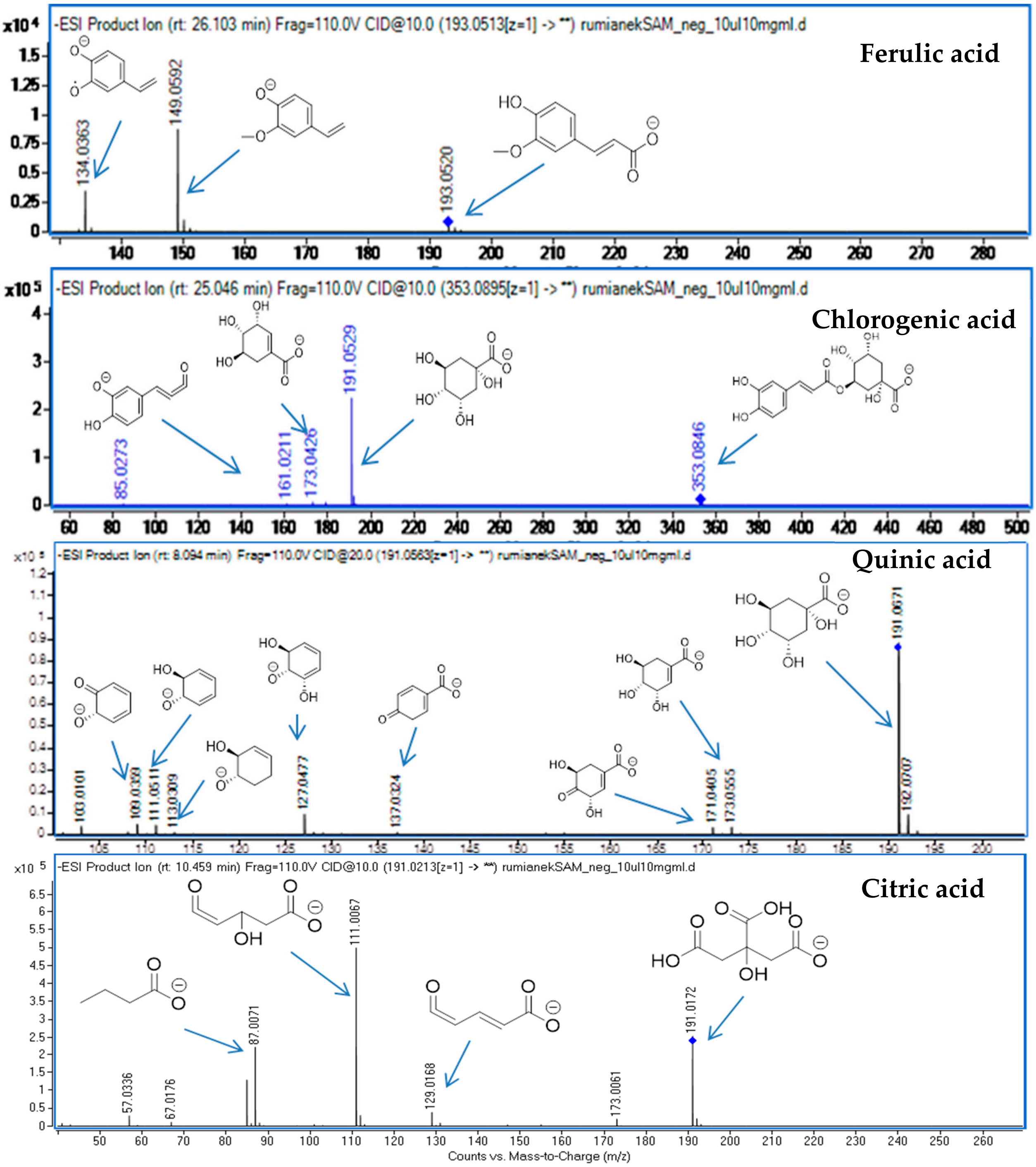

Supplement: Supplementary file 1 [file molecules-30-01979-s001.zip › molecules-3564126-supplementary.pdf]
